# Neuroprotective and Cognitive Enhancement Potentials of *Angelica gigas* Nakai Root: A Review

**DOI:** 10.3390/scipharm85020021

**Published:** 2017-04-28

**Authors:** Kandhasamy Sowndhararajan, Songmun Kim

**Affiliations:** School of Natural Resources and Environmental Sciences, Kangwon National University, Chuncheon 24341, Korea; sowndhar1982@gmail.com

**Keywords:** *Angelica gigas*, cognitive, decursin, neuroprotective, decursinol

## Abstract

*Angelica gigas* Nakai is an important medicinal plant with health promoting properties that is used to treat many disorders. In traditional herbal medicine, the root of this plant is used to promote blood flow, to treat anemia, and is used as sedative or tonic agent. The root contains various bioactive metabolites; in particular, decursin and decursinol (pyranocoumarin type components) have been reported to possess various pharmacological properties. Recently, several in vitro and in vivo studies have reported that the crude extracts and isolated components from the root of *A. gigas* exhibited neuroprotective and cognitive enhancement effects. Neuronal damage or death is the most important factor for many neurodegenerative diseases. In addition, recent studies have clearly demonstrated the possible mechanisms behind the neuroprotective action of extracts/compounds from the root of *A. gigas*. In the present review, we summarized the neuroprotective and cognitive enhancement effects of extracts and individual compounds from *A. gigas* root.

## 1. Introduction

The age-related neurodegenerative disorders including Alzheimer’s disease, Parkinson’s disease, Huntington’s disease, and multiple sclerosis are caused by progressive loss of structure or function of neurons, resulting in neuronal cell death. The World Health Organization (WHO) has stated that neurodegenerative diseases will be the second leading cause of natural death by 2040 [1]. In recent times, considerable attention has focused on understanding the molecular mechanism of neuronal cell death to develop strategies for controlling or delaying the process of neurodegenerative diseases. A number of neurotoxic factors such as oxidative stress, inflammatory cytokines, abnormal protein dynamics and mitochondrial dysfunction are mainly responsible for neuronal damage [2,3,4]. Among them, reactive oxygen species (ROS)-induced oxidative stress is a major factor in neuronal cell death by causing oxidative damage to DNA, proteins, and lipids. Further, oxidative stress is mainly related to secondary cell death in many central nervous system disorders [5,6]. Apoptosis also plays an important role in neuronal cell death. Previous studies reported that two major signaling pathways contributed to apoptotic type cell death, i.e., intrinsic (mitochondrial) and extrinsic (death receptor) pathways [7]. In addition, several transcription factors play a major role in neuronal cell death. Among them, nuclear factor erythroid-derived 2 (NF-E2) related factor (Nrf2), cAMP-response element binding protein (CREB), mitogen-activated protein kinases (MAPKs), nuclearfactor-kappa B (NF-κB), janus kinase/signal transducer and activator of transcription, Wnt and Toll like receptor-4 are key players in the protection against neuronal damage. These transcription factors are mainly associated with the activation of antioxidant enzymes, differentiation and adaptation of cells and regulate the expression of various genes mediating the inflammatory response [8,9,10]. In addition, they play a pivotal role in the normal development of neurons and protection against neuronal homeostasis, axonogenesis and synaptic plasticity [4,11].

In this connection, the main aim of neuroprotective agents is to protect the central nervous system against the damage of neuronal cells [12,13,14]. A variety of treatment methods are available to control neurodegenerative diseases, such as dopaminergic treatments, cholinesterase inhibitors, antipsychotic drugs, and brain stimulation [15,16,17,18]. Further, riluzole, non-steroidal anti-inflammatory drugs, caffein A2A receptor antagonists and CERE-120 (adeno-associated virus serotype 2-neurturin) have been used to cure neurodegenerative diseases [19]. Although these drugs provide some promising results, they produce several adverse effects in long-term use. Further, currently available medications for the treatment of neurodegenerative diseases are able to give only symptomatic relief for the patients. Hence, it is essential to develop safe, multi-targeted and more effective drugs to treat neurodegenerative diseases. In the past decades, there has been growing interest in developing neuroprotective agents from natural products to prevent the damage or death of neuronal cells. Previously, a number of studies reported that natural products, especially from plant sources, markedly showed a neuroprotective effect. Plant products (crude extracts and pure compounds) possess versatile bioactive properties and could be useful to improve human health and protect against neurodegeneration [14,20]. Although many phytodrugs exhibit remarkable biological activities, most of the studies ended only in patent applications, article publications, and report preparations. To ensure the safety, efficacy and quality of herbal drugs, well-controlled and randomized clinical trials are still required in order to prove their exact action. Performing clinical trials using plant-based drugs is very challenging because these trials require large participant groups, long durations, and expensive healthcare services; they have ethical issues, and it is difficult to obtain enough funds [21,22].

The genus *Angelica* L. belongs to the family of Apiaceae (Umbelliferae) that contains about 60 species of biennial or short lived perennial herbs and occurs widely in Asia, Europe and North America. According to its area of distribution, three common species of *Angelica* species, *A. gigas*, *A. sinensis* and *A. acutiloba*, are found in Korea, China and Japan, respectively [23,24]. In Korean traditional medicine, the dried root of *A. gigas* has been mainly used as a treatment for anemia and as a sedative or a blood tonic agent [25]. Previous studies reported that the *A. gigas* root has various pharmacological properties such as anticancer, antibacterial, immune-stimulating, antiplatelet aggregation, neuroprotective, anti-inflammatory, antinematodal, and antioxidant properties [24]. The root is comprised of several bioactive components such as pyranocoumarins, essential oils, and polyacetylenes. In the root of *A. gigas*, decursin is the most abundant pyranocoumarin compound with a wide range of pharmacological properties [26]. Recent scientific reports demonstrated that the crude extracts (herbal mixture, methanol, ethanol and water) and isolated compounds (decursin, decursinol, decursinol angelate and nodekenin) from the root of *A. gigas* showed remarkable neuroprotective effects against various toxic agents under in vitro and in vivo conditions [27,28]. Previous studies clearly revealed that the crude extracts and isolated components from the root of *A. gigas* exert neuroprotective activity by regulating numerous biological processes [29,30,31].

**Figure 1:**
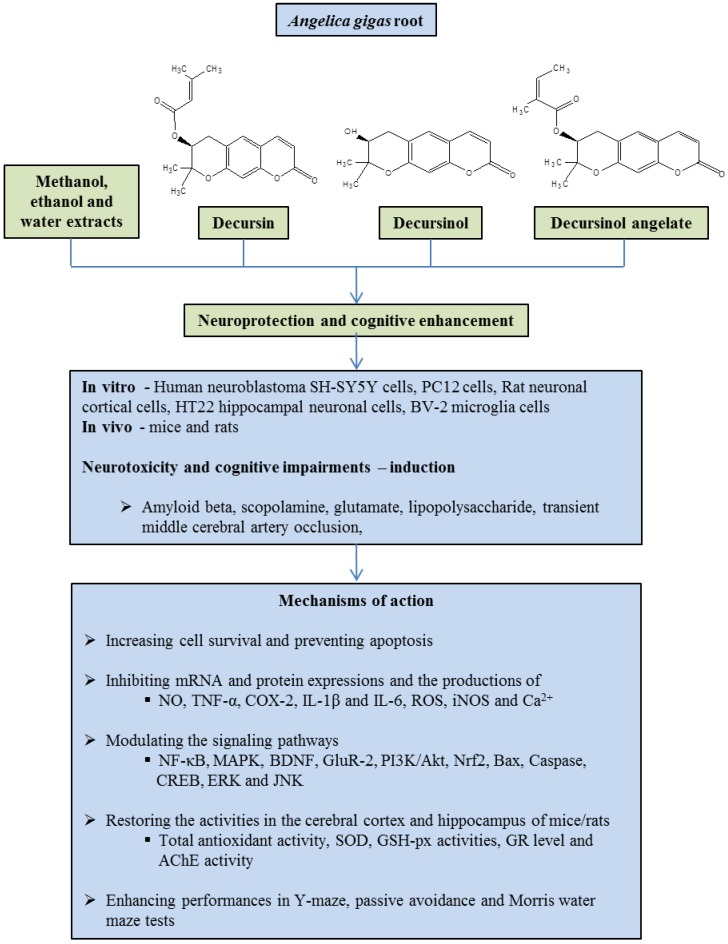
Neuroprotective effect of extracts and important components from the root of *Angelica gigas*. NO: nitric oxide; TNF-α: tumor necrosis factor-α; COX-2: cyclooxygenase-2; IL: interleukin; ROS: reactive oxygen species; iNOS: inducible nitric oxide synthase; NF-κB: nuclear factor-kappa B; MAPK: mitogen-activated protein kinases; BDNF: brain-derived neurotrophic factor; GluR-2: glutamate receptor-2; PI3K: phosphatidylinositol 3-kinase; CREB: cAMP-response element binding protein; ERK: extracellular signal–regulated kinase; JNK: c-Jun amino-terminal kinase; SOD: superoxide dismutase; GSH-px: glutathione peroxidase; GR: glutathione reductase; AChE: acetylcholinesterase.

In recent times, controlling neuronal damage and understanding neuroprotective mechanisms have been the main interests of neuroscientists. In the present review, we summarized the current knowledge in regards to neuroprotective and cognitive enhancement activities of *A. gigas* root (Table 1 and Figure 1). In addition, this review provides appropriate information in relation to the molecular mechanisms behind the neuroprotective action of *A. gigas* root against various neurotoxic agents.

## 2. Neuroprotective Properties of *A. gigas* Root

### 2.1. Extracts

ESP-102 is a standardized extract of herbal mixture, comprised of 70% ethanol extract from *A. gigas* root, and 100% ethanol extract from the fruits of *Saururus chinensis* and *Schisandra chinensis*. The ratio of these three different components was adjusted to 8:1:1 (*A. gigas*: *S. chinensis*: *S. chinensis*). In Korea, ESP-102 has been used as an important herbal medicine and dietary supplement. Acute oral treatment (single administration) and prolonged oral daily treatments (10 days) of mice with ESP-102 significantly improved scopolamine-induced memory deficits based on the passive avoidance and Morris water maze tests. Further, ESP-102 significantly protected cortical neuronal cells against glutamate- or Aβ_25–35_-induced neurotoxicity [32]. Ma et al. [33] also reported that ESP-102 significantly improved scopolamine-induced memory impairment in mice and protected against glutamate-induced toxicity in rat cortical cells. In the glutamate-induced toxicity in neuronal cells, ESP-102 decreased the intercellular calcium concentration ([Ca^2+^]i) and inhibited the overproduction of nitric oxide (NO) and ROS. ESP-102 also maintained the level of enzymatic antioxidants such as superoxide dismutase, glutathione peroxidase (GSH-px) and glutathione reductase (GR). Furthermore, ESP-102 controlled the loss of mitochondrial membrane potential in glutamate-induced rat cortical cells. Recently, the neuroprotective effect of ESP-102 against scopolamine-induced toxicity in rat hippocampal slice was studied by Kim et al. [34]. The results showed that ESP-102 competitively antagonized the preventative long-term potentiation effect in the scopolamine-induced hippocampal slice. ESP-102 also rescued the reduction of brain-derived neurotrophic factor (BDNF) and GluR-2 expression in scopolamine-induced tissue. Based on the results, ESP-102 can be used as an effective herbal ingredient for the treatment of neuronal damage and memory impairments.

In traditional medicine, Jangwonhwan (boiled extract contains 12 medicinal herbs/mushroom) has been prescribed for patients with cognitive dysfunction. Recently, a modified recipe of Jangwonhwan (LMK02) consisting of 7 medicinal plants/mushroom (red *Panax ginseng* (root, 20 g), *Acorus gramineus* (rhizome, 16 g), white *Poria cocos* (sclerotium, 16 g), *A. gigas* (root, 12 g), *Ophiopogon japonicas* (rhizome, 12 g), *Scrophularia buergeriana* (root, 16 g) and *Thuja orientalis* L. (seed, 12 g)) was shown to have therapeutic potential to ameliorate AD-like pathology. LMK02 also significantly protected against neuronal damage in H19-7 cells of rat hippocampus caused by Aβ-induced neurotoxicity. In H19-7 cells, LMK02 inhibited apoptosis and ROS production [35]. LMK03 is another modified recipe of Jangwonhwan consisting of white *Poria cocos* sclerotium and *A. gigas* root. Seo et al. [36,37] examined the effect of LKM02 and LKM03 on Aβ deposition in the brain of Tg-APPswe/PS1dE9 mice. When compared with LKM03, LMK02 efficiently reduced the levels of Aβ_1–42_ and Aβ_1–40_ along with a reduction in plaque deposition in the brain of Tg-APPswe/PS1dE9 mice. The authors reported that LMK02 partially suppressed the accumulation of oxidative stress and prevented the down-regulation of phospho-CREB and calbindin. In the in vitro study, LMK02 effectively inhibited oxidative stress and Aβ-induced neurotoxicity in SH-SY5Y neuroblastoma cells. These results suggested that LMK02 has therapeutic potential to ameliorate AD-like pathology in the brain of Tg-APPswe/PS1dE9 mice.

Bozhougyiqi-Tang (BZYQT), a traditional herbal medicine (*Panax ginseng* (3.75 g), *Astragalus membranaceus* (5.63 g), *A. gigas* (1.88 g), *Bupleurum falcatum* (1.13 g), *Citrus unshiu* (1.88 g), *Glycyrrhiza uralensis* (3.75 g), *Atractylodes japonica* (3.75 g) and *Clematis heracleifolia* (1.13 g)), has been therapeutically used for the treatment of pulmonary tuberculosis. Weon et al. [38] examined the neuroprotective effect of fermented BZYQT and unfermented BZYQT in HT22 cells. The fermented BZYQT exhibited higher neuroprotective activity against glutamate-induced neurotoxicity in HT22 cells than unfermented BZYQT. Additionally, the fermented BZYQT significantly enhanced the cognitive performances in passive avoidance and Morris water maze tests. The herbal mixtures ESP-102, LMK02, LMK03 and BZYQT exhibited remarkable neuroprotective potential in vitro as well as in vivo models, and their activities could be described as a synergistic effect of combined plant extracts.

The water extract from the hairy root of *A. gigas* showed neuroprotective activity in transient middle cerebral artery occlusion-induced focal cerebral ischemia rats. The results revealed that the water extract significantly reduced the brain infarction volumes and edema in rats. It also decreased the blood-brain-barrier permeability and neuronal death as well as inhibited the activation of astrocytes and microglia in ischemic brains. Further, this extract significantly increased the expression of angiopoietin-1 (Ang-1), tight junction proteins (ZO-1 and Occludin) and vascular endothelial growth factor (VEGF) through the activation of phosphorylation of phosphatidylinositol 3-kinase (PI3K)/AKT pathway. In ischemic brains, this extract also significantly increased the intercellular adhesion molecule-1 (ICAM-1) expression [29].

In traditional herbal medicine, INM-176 is a standardized ethanolic extract (80%) of *A. gigas* that has been used in China, Japan, and Korea as a treatment for anemia or as a sedative. Park et al. [40,41] investigated the effect of INM-176 on scopolamine- or Aβ_1–42_-induced memory impairment in mice and lipopolysaccharide (LPS)-induced neuronal damage in primary microglial cells and mice. INM-176 significantly attenuated the scopolamine- or Aβ_1–42_ or LPS-induced cognitive deficit in the passive avoidance and the Morris water maze tasks. Further, INM-176 inhibited acetylcholinesterase (AChE) activity in the hippocampal tissue in vitro and ex vivo. In addition, INM-176 attenuated Aβ_1–42_- or LPS-induced astrocyte activation in the hippocampus region of mice. INM-176 also effectively inhibited the production of NO and suppressed the expressions of tumor necrosis factor-α (TNF-α) and interleukin-1β (IL-1β), inducible nitric oxide synthase (iNOS), cyclooxygenase-2 (COX-2) in LPS-induced primary microglial cells. These results suggest that INM-176 has remarkable neuroprotective and cognitive enhancement effects against various neurotoxic agents. The ethanol extract of *A. gigas* root also significantly blocked Aβ_1–42_-induced memory impairment in the passive avoidance test [25].

A comparative neuroprotective effect of different parts (root head, root body and hairy root) of *A. gigas* in middle cerebral artery occlusion-induced ischemic rats and LPS-induced BV2 microglia was investigated by Shin and Park [30]. Among the different parts of root, a 30% ethanol extract of hairy root significantly reduced infarction volume in ischemic brains and also inhibited the expression of iNOS, bax and caspase-3. The hairy root extract remarkably inhibited the production of inflammatory mediators such as NO, TNF-α and IL-6 in BV2 cells and suppressed the expression of iNOS and COX-2. Furthermore, the hairy root extract suppressed LPS-induced phosphorylation of extracellular signal–regulated kinase (ERK1/2) and c-Jun amino-terminal kinase (JNK) MAPK in BV2 cells. The methanol extract (80%) of *A. gigas* root exhibited strong neuroprotective activity in 4-vessel occlusion-induced global ischemia rats by attenuating COX-2 induction in the hippocampus [39]. In another study, Piao et al. [26] found that oral solid formulations of *A. gigas* and Soluplus obtained from a hot-melting extrusion method showed a higher cognitive enhancement effect than ethanol extract in scopolamine-induced memory-impaired mice.

### 2.2. Decursin

The root of *A. gigas* has various pharmacological properties, and most these activities are mainly attributed to the major active component decursin. Decursin has been reported in some herbal formulas for the treatments of obesity, inflammation, fever, amnesia, neuralgia, rheumatism, hyperlipidemia and other diseases. Due to the hydrophobicity of decursin, this compound can be extracted using ethanol or supercritical carbon dioxide fluid [48]. Decursin induces apoptosis in various human cancer cells including prostate, breast, bladder and colon cancer cells. Further, decursin inhibits NF-κB activation in macrophages and cancer cells [49]. Among the various biological properties, decursin is also a potent neuroprotective agent and an effective cognitive enhancer. Decursin significantly ameliorated scopolamine-induced amnesia in mice measured in both the passive avoidance and the Morris water maze tests. The results revealed that decursin may exert anti-amnestic activity through the inhibition of AChE activity in the hippocampus of mice [42]. Kang and Kim [43] found that decursin showed neuroprotective activity in rat cortical cells against glutamate-induced oxidative stress by reducing calcium influx and acting on the cellular antioxidative defence system.

Li et al. [44] reported that decursin significantly decreased cytotoxicity and lipid peroxidation and increased glutathione contents and antioxidant enzyme activities in Aβ-induced neurotoxicity in PC12 cells. Furthermore, decursin suppressed Aβ aggregation and increased Nrf2 expression in PC12 cells. Li et al. [27] reported that decursin protected PC12 cells against Aβ_25−35_-induced oxidative cytotoxicity through intrinsic free radical scavenging activity and activation of MAPK pathways that lead to the activation of Nrf2 and induction of HO-1. Furthermore, selective neuronal death, astrogliosis, and oxidative stress were strongly inhibited by decursin. Li et al. [31] reported that decursin significantly inhibited Aβ_25–35_-induced cytotoxicity and apoptosis in PC12 cells by reducing the mitochondrial membrane potential, inhibiting ROS production, and decreasing the mitochondrial release of cytochrome c. Furthermore, decursin effectively suppressed caspase-3 activity and moderated the ratio of Bcl-2/Bax in Aβ_25–35_-induced PC12 cells.

### 2.3. Decursinol and Decursinol Angelate

The long-term oral administration (4 weeks) of decursinol significantly attenuated Aβ_1–42_-induced memory impairment in mice based on the passive avoidance performance and Y-maze test [25]. Kang and Kim [43] reported that decursinol effectively protected against glutamate-induced neurotoxicity in cortical cells by reducing [Ca^2+^]i. In addition, decursinol showed higher neuroprotective effect against KA-induced neurotoxicity than N-methyl-D-aspartate-induced toxicity in cortical neurons. Moreover, decursinol significantly increased the glutathione level and GSH-px activity and efficiently decreased the overproduction of cellular peroxide in glutamate-injured cortical cells. In addition, the protective effect of decursinol angelate on Aβ-induced neurotoxicity in the rat PC12 cells was reported by Li et al. [44]. Pretreatment of PC12 cells with decursinol angelate effectively decreased cytotoxicity and lipid peroxidation. Further, decursinol angelate increased the glutathione level, antioxidant enzyme activities as well as the expression of Nrf2 in Aβ-induced PC12 cells. Zhang et al. [50] reported that decursin and decursinol angelate are rapidly converted into decursinol in rodents after oral administration.

### 2.4. Other Compounds

In the *A. gigas* root, 13 new dihydropyranocoumarins were isolated using neuroprotective activity-guided fractionation. Among them, 4“-hydroxytigloyldecursinol, 4“-hydroxydecursin, (2”S,3”S)-epoxyangeloyldecursinol, and (2”R,3”R)-epoxyangeloyldecursinol, marmesinin, nodakenin and columbianetin-O-β-D-glucopyranoside showed strong neuroprotective activity against glutamate-induced neurotoxicity in rat cortical cells. In the structure-activity relationship of these coumarins, the authors suggested that the cyclization of the isoprenyl group (dihydropyran or dihydrofuran) or the furan ring at the C-6 position of coumarin and lipophilicity played a major role in the protection of neurons [46,47]. In another study, nodakenin significantly enhanced the scopolamine-induced cognitive decline in the passive avoidance, Y-maze and Morris water maze tests. Further, nodakenin effectively inhibited AChE activity in a dose-dependent manner [45].

## 3. Conclusions

It is well known that herbal extracts/individual components can considerably contribute to protection against neuronal damage through various modes of action. In this review, we highlighted the neuroprotective and cognitive enhancement properties of *A. gigas* and their modes of action. The published reports revealed that the extracts and isolated components from the root of *A. gigas* showed neuroprotective and cognitive enhancement properties through various mechanisms such as anti-apoptosis, antioxidative actions, inhibiting mRNA and protein expressions of inflammatory mediators and regulating a number of signaling pathways. Hence, this review will offer a scientific basis for future studies in relation to detailed molecular mechanisms of the neuroprotective action of natural products. In conclusion, the *A. gigas* root can serve as an effective neuroprotective agent by modulating various pathophysiological processes. Due to the multi-targeted actions of these coumarin-type components, they could represent a promising natural product to develop new and safe neuroprotective drugs.

## Figures and Tables

**Table 1:** Neuroprotective and cognitive enhancement properties of compounds and extracts from the root of *Angelica gigas*.

Compound/Extract	Model	Mechanism	Dose	References
ESP-102 (a combined ethanol extract, consists of *A. gigas*, *Saururus chinensis* and *Schisandra chinensis*)	Scopolamine-induced memory impairment in mice. Aβ_25–35_ or glutamate-induced neurotoxicity in cortical neurons of rats.	Enhances cognitive performances in the passive avoidance and Morris water maze tests. Protects against neuronal damage.	Mice: 1 to 100 mg/kg; Cell line: 0.001, 0.01 and 0.1 μg/mL	[32]
	Glutamate-induced toxicity in rat cortical cells.	Decreases [Ca^2+^]i, inhibits the production of NO and ROS. Increases SOD, GSH-px and GR. Maintains mitochondrial membrane potential.	0.5 mg/mL to 50 mg/mL	[33]
	Scopolamine-induced memory impairment in rat hippocampus tissue	Antagonizes the preventative long-term potentiation effect. Increases BDNF and GluR-2 expressions. Acts on the AMPA/NMDA receptors.	10 μg/mL	[34]
LMK02 - Jangwonhwan (a herbal mixture of boiled extracts including *A. gigas*)	Aβ-induced neurotoxicity in H19-7 cells from rat hippocampus.	Protects against cytotoxicity. Inhibits formation of Aβ oligomer. Activates anti-apoptosis and decreases the production of ROS.	1 to 100 μg/mL	[35]
	Aβ-induced neurotoxicity in SH-SY5Y cells and Tg-APPswe/PS1dE9 mice.	Protects against cytotoxicity. Reduces Aβ_1–42_ and Aβ_1–40_ levels and β-amyloid plaque deposition in the brain.	Mice: 400 mg/kg, SH-SY5Y cells: 50 to 150 mg/mL	[36]
LMK03-Jangwonhwan (a combined boiled extract, consists of white *Poria cocos* and *A. gigas*)	Aβ-induced neurotoxicity in SH-SY5Y neuroblastoma cells and Tg-APPswe/PS1dE9 transgenic mice.	Protects against cytotoxicity. Reduces Aβ_1–42_ and Aβ_1–40_ levels and β-amyloid plaque deposition in the brain.	Mice: 300 mg/kg, SH-SY5Y cells: 50 and 100 mg/mL	[37]
Fermented Bozhougyiqi-Tang (Herbal mixture) containing *A. gigas*	Scopolamine-induced memory impairments in mice and glutamate induced neurotoxicity in HT22 cells.	Protects against cytotoxicity. Enhances cognitive performance in the Morris water maze test.	Mice: 30, 100 and 200 mg/kg HT22 cells: 10, 100 and 1000 μg/mL	[38]
Water extract of *A. gigas* root	Transient middle cerebral artery occlusion-induced focal cerebral ischemia in rats.	Decreases the brain infarction volumes and edema. Decreases the blood brain barrier permeability and neuronal death and inhibits the activation of astrocytes and microglia. Increases the expression of Ang-1, Tie-2, VEGF, ZO-1 and Occludin via activation of the PI3K/Akt pathway. Increases the expression of ICAM-1.	10, 25, 50 and 100 mg/kg	[29]
Methanol extract of *A. gigas* root	Four-vessel occlusion-induced ischemia in rats.	Attenuates COX-2 induction in hippocampus.	100, 250 and 500 mg/kg	[39]
Ethanol extract of *A. gigas* root	Aβ-induced memory impairment in mice.	Enhances cognitive performances in the passive avoidance performance and Y-maze tests.	Ethanol extract: 0.1%	[25]
	Scopolamine/Aβ-induced cognitive dysfunction in mice.	Enhances cognitive performances in the passive avoidance and Morris water maze tests. Inhibits AChE activity. Attenuates the astrocyte activation and cholinergic neuronal damage in the hippocampus and the nucleus basalis of Meynert.	150, 300, 600 and 1200 mg/kg	[40]
	LPS-induced neuronal injury in BV2 microglial cells and mice.	Inhibits NO release and suppressed the expressions of TNF-α and IL-1β, iNOS and COX-2. Attenuates neuronal damage in a hippocampal slice culture. Enhances cognitive performances in the passive avoidance and Y-maze tests. Suppresses the activation of microglia or astrocytes.	0.05–2 μg/mL	[41]
Ethanol extract of *A. gigas* root	Neuronal death in transient middle artery occlusion/reperfusion-induced ischemic rats and LPS-induced inflammatory response in BV2 microglia.	Decreases infarction volume in ischemic brains and inhibits the expression of iNOS, bax and caspase-3. Inhibits the production of NO, TNF-α and IL-6, and suppresses the expression of iNOS and COX-2. Attenuates phosphorylation of ERK1/2 and JNK MAPK in BV2 cells.	50 and 100 mg/kg	[30]
Hot-melting extrusion -processed *A. gigas*/Soluplus	Scopolamine-induced memory disruption in mice.	Enhances cognitive performances in the Morris water maze and passive avoidance tests.	200 mg/kg	[26]
Decursin	Scopolamine-induced amnesia in mice.	Enhances cognitive performances in the passive avoidance and Morris water maze tests. Inhibits AChE activity.	1 and 5 mg/kg	[42]
	Glutamate-induced toxicity in rat cortical cells.	Protects against cytotoxicity. Reduces [Ca^2+^]i in cortical cells. Increases the glutathione and GSH-px levels.	0.1–10.0 μM	[43]
Decursin	Aβ-induced neurotoxicity in PC12 cells.	Increases Nrf2 expression and suppresses the aggregation of Amyloid-β.	0.01–10.0 μM	[44]
	Aβ-induced neurotoxicity in PC12 cells.	Induces Nrf2 nuclear translocation, the upstream of HO-1 expression, ERK and dephosphorylated p38.	0.01–10 μM	[27]
	Aβ-induced neurotoxicity in PC12 cells.	Protects against cytotoxicity and apoptosis. Reduces the mitochondrial membrane potential, ROS production, and mitochondrial release of cytochrome c. Suppresses the caspase-3 activity and moderated the ratio of Bcl-2/Bax.	0.01–10 μM	[31]
Decursinol	Aβ-induced memory impairment in mice.	Enhances cognitive performances in the passive avoidance and Y-maze tests.	0.001%, 0.002%, and 0.004%	[25]
	Glutamate-induced toxicity in rat cortical cells.	Protects against cytotoxicity. Reduces [Ca^2+^]i in cortical cells. Protects neurons against KA-induced neurotoxicity. Increases the glutathione and GSH-px levels.	0.1–10.0 μM	[43]
Decursinol angelate	Aβ-induced neurotoxicity in PC12 cells.	Increases Nrf2 expression and suppresses the aggregation of Aβ.	0.1–10.0 μM	[44]
Nodakenin	Scopolamine-induced memory disruption in mice.	Enhances cognitive performances in the passive avoidance, Y-maze and Morris water maze tests. Inhibits AChE activity.	10 mg/kg	[45]
	Glutamate-induced toxicity in rat cortical cells.	Protects against cytotoxicity.	0.1 to 10 μM	[46]
4″-Hydroxytigloyldecursinol, 4″-hydroxydecursin, (2″S,3″S)-epoxyangeloyldecursinol, (2″R,3″R)-epoxyangeloyldecursinol, Marmesinin, columbianetin-O-β-D-glucopyranoside	Glutamate-induced toxicity in rat cortical cells.	Protects against cytotoxicity.	0.1 to 10 μM	[46,47]

[Ca^2+^]i: intracellular calcium; AMPA/NMDA: α-amino-3-hydroxy-5-methyl-4-isoxazolepropionic acid/N-methyl-D-aspartate; VEGF: vascular endothelial growth factor; ZO-1: zonula occludens-1; ICAM-1: intercellular adhesion molecule-1; HO-1: heme oxygenase-1.
